# Generating Lifetime-Enhanced Microbubbles by Decorating Shells with Silicon Quantum Nano-Dots Using a 3-Series T-Junction Microfluidic Device

**DOI:** 10.1021/acs.langmuir.2c00126

**Published:** 2022-08-26

**Authors:** Bingjie Wu, C. J. Luo, Ashwin Palaniappan, Xinyue Jiang, Merve Gultekinoglu, Kezban Ulubayram, Cem Bayram, Anthony Harker, Naoto Shirahata, Aaqib H. Khan, Sameer V. Dalvi, Mohan Edirisinghe

**Affiliations:** † Department of Mechanical Engineering, University College London (UCL), London WC1E 7JE, U.K.; ‡ Department of Physics and Astronomy, University College London (UCL), London WC1E 7JE, U.K.; § Department of Basic Pharmaceutical Sciences, Faculty of Pharmacy, Hacettepe University, Ankara 06100, Turkey; ∥ Nanotechnology and Nanomedicine Division, Institute for Graduate Studies in Science & Engineering, Hacettepe University, Ankara 06100, Turkey; ⊥ WPI International Center for Materials Nanoarchitectonics (MANA), National Institute for Materials Science (NIMS), 1-1 Namiki, Tsukuba, Ibaraki 305-0044, Japan; # Graduate School of Chemical Sciences and Engineering, Hokkaido University, Sapporo 060-0814, Japan; ¶ Chemical Engineering, Indian Institute of Technology Gandhinagar, Palaj, Gandhinagar 382355, Gujarat, India

## Abstract

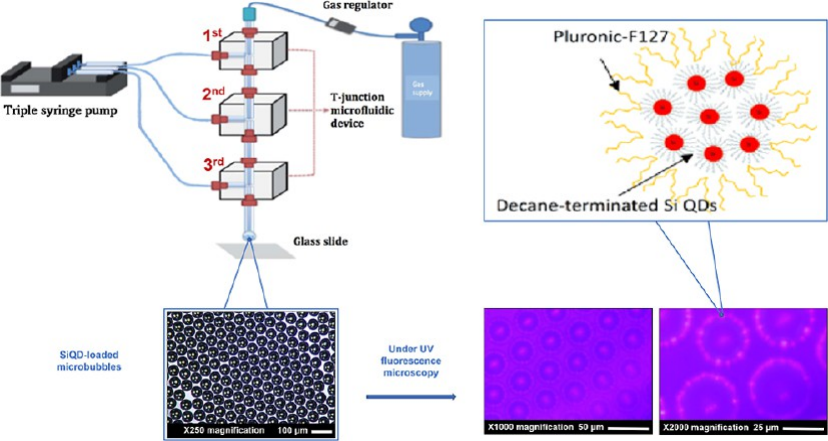

Long-term stability of microbubbles is crucial to their effectiveness. Using a new microfluidic device connecting three T-junction channels of 100 μm in series, stable monodisperse SiQD-loaded bovine serum albumin (BSA) protein microbubbles down to 22.8 ± 1.4 μm in diameter were generated. Fluorescence microscopy confirmed the integration of SiQD on the microbubble surface, which retained the same morphology as those without SiQD. The microbubble diameter and stability in air were manipulated through appropriate selection of T-junction numbers, capillary diameter, liquid flow rate, and BSA and SiQD concentrations. A predictive computational model was developed from the experimental data, and the number of T-junctions was incorporated into this model as one of the variables. It was illustrated that the diameter of the monodisperse microbubbles generated can be tailored by combining up to three T-junctions in series, while the operating parameters were kept constant. Computational modeling of microbubble diameter and stability agreed with experimental data. The lifetime of microbubbles increased with increasing T-junction number and higher concentrations of BSA and SiQD. The present research sheds light on a potential new route employing SiQD and triple T-junctions to form stable, monodisperse, multi-layered, and well-characterized protein and quantum dot-loaded protein microbubbles with enhanced stability for the first time.

## Introduction

Microbubbles, gas-filled structures characterized by a core–shell composition, provide an excellent platform for a wide range of key applications such as mineral processing,^1,2^ water-purification and environmental engineering,^3−6^ biosensing,^7,8^ food engineering,^9,10^ and biomedical engineering.^11,12^ Different microbubble sizes are used in these operations, but imparting stability, that is negligible change in diameter over prolonged periods, is a crucial feature in them all. Accordingly, precise production control of the size and high lifetime stability of the microbubbles are essential. The determination of microbubble stability is related to the elasticity and permeability of the shell substance. Therefore, stability can be enhanced by manipulating shell composition such as material properties, concentrations, and shell thickness. However, it must be noted that gas solubility is also a major factor determining microbubble stability and was kept to one filling gas (nitrogen) in this work.

Bovine serum albumin (BSA) is used in this work as the model shell material. BSA is a cost-effective material to form protein microbubbles while allowing adsorption of various nanoparticles and biomolecules. Previous research has shown that adsorption of solid nano-dots/nanoparticles on the microbubble shell can enhance bubble lifetime by resisting shell compression during dissolution through particle jamming at the solid–gas interface.^7^ In this work, photoluminescent silicon quantum nano-dots (SiQD) were synthesized and loaded on the BSA shell to explore their potential as a stabilizer for enhancing microbubble lifetime. SiQD have excellent photoluminescence quantum yield, photostability, low toxicity, biocompatibility, and good water dispersion,^13,14^ making them a versatile material as biomarkers, drug delivery tracking agents, contrast agents, and intracellular probes for microbubble imaging and drug delivery in both photoacoustic and ultrasound imaging.^15,16^

Many methods have been employed to produce microbubbles, which include not only sonication^17^ but also high shear emulsification,^18^ coaxial electro-hydrodynamic atomization,^19^ and pressurized gyration.^7^ A key challenge with each of these methods is the broad size distribution of the microbubbles generated.^19^ In contrast, the microfluidic method used in this work enables precise control over the size and polydispersity of the microbubbles generated. Moreover, the flow-focusing microfluidic geometry is a common technique adopted in fabrication of gas-filled microbubbles.^20^ In this case, microbubble diameter, velocity, and frequency of generation can be altered by the gas–liquid flow rate ratio, material viscosities, and orifice dimension.^20,21^ Because the dispersed phase is confined in the central region of the main channel, a flow-focusing device, which is comparable with T-junction microchannels, generally produces spherical rather than plug-like microbubbles.^22^ Therefore, it is possible to protect the microbubbles from shear forces, and from contacting wall channels, which will lead to the damage or adhesion of the microbubbles. However, T-junction cross-flow devices are among the simplest and the most robust and dependable geometries to reproducibly generate highly monodisperse microbubbles with coefficient of variation less than 2%.^23,24^ Therefore, for the first time in microbubble research, this work focuses on developing a triple T-junction microfluidic system comprising three capillary T-junctions, embedded with relatively large diameter capillaries (200 and 100 μm diameter) connected in series to produce monodisperse microbubbles with enhanced stability. The large diameter of the capillary channels has the advantage of easy manual assembly and handling, low risk of material blockages, and low likelihood of breakage, ensuring continuous production.

We generate stable monodisperse microbubbles loaded with photofluorescent quantum nano-dots and systematically characterize the effect of key parameters in the process in terms of the number of T-junctions in series, capillary diameter, BSA and SiQD concentration, liquid flow rate, and gas pressure on the size and stability of the microbubbles. The stability of the SiQD-loaded microbubbles is quantified and modeled, and thereby theoretical and experimental predictions for stability are compared.

## Materials and Experimental Methods

### Materials

Food grade BSA (>96% lyophilized powder free of fatty acid and globulin, molecular weight *M*_w_ = 66 kDa, Sigma-Aldrich, UK) was used as received. Dispersion (100 μg mL^–1^) of double-shelled decane-terminated SiQD coated with Pluronic F127 in Milli-Q water was prepared and characterized as reported earlier by Chandra et al.^13,14^

### Solution Preparation and Characterization

**Figure 1:**
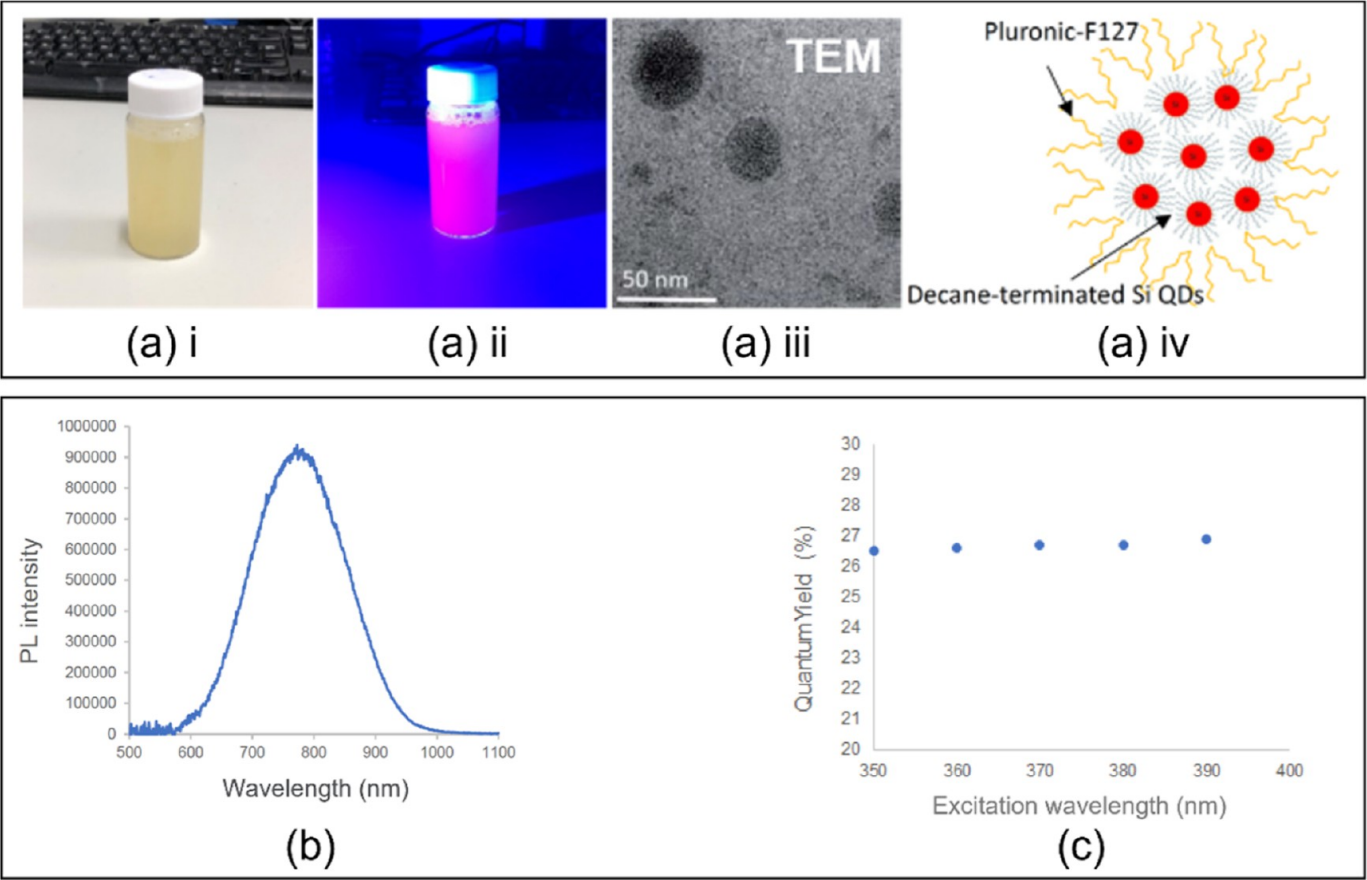
(a) SiQD dispersed in Milli-Q water under (a) (i) white light and (ii) UV light; (iii) transmission electron micrograph (TEM) of SiQD; (iv) schematic representation of the molecular structure of double-shelled decane-terminated Pluronic F127 coated SiQD. (b) Photoluminescence spectrum excited at 390 nm (UV light) giving red-photoluminescence at 780 nm. (c) Photoluminescence quantum yields (>26%) excited at 350–390 nm.

BSA solutions (5, 10, 15 wt %) were prepared by dissolving BSA in distilled/deionized water at room temperature (∼23 °C) with continuous stirring for 2 h using a magnetic stirrer. SiQD dispersion (100 μg mL^–1^, Figure 1) was diluted in distilled/deionized water to obtain SiQD dispersions of varying concentrations (1, 5, 10, 25, and 50 μg mL^–1^). For SiQD decorated microbubbles, SiQD dispersions were mixed with BSA solution by gentle magnetic stirring at ambient temperature as described above to generate 10 wt % BSA solutions containing 1, 5, 10, 25, 50, and 100 μg mL^–1^ SiQD. Solutions were preserved at 4 °C and used within 24 h.

The surface tension, density, and viscosity of the BSA solutions with and without SiQD were characterized subsequent to preparation. A tensiometer (Kruss K9, model DSA100, Kruss GmbH, Hamburg, Germany) was used to measure the static surface tension of the solution (Wilhelmy’s plate method). A standard density bottle (DIN ISO 3507-Gay-Lussac) was used to measure solution density. Viscosity was measured with a rheometer (BrookField DV-11 Ultra programmable Rheometer). All the equipment were calibrated with distilled/deionized water prior to use at a relative humidity of 40%. All measurements (Table 1) were carried out at room temperature (∼23 °C).

**Table 1:** Physical Properties of Solutions Used in the Experiments

aqueous solution	viscosity (mPa s)	surface tension (mN m^–1^)	density (kg m^–3^)
5 wt % BSA	1.21 ± 2.12	51.3 ± 2.8	1037 ± 3.7
10 wt % BSA	1.43 ± 0.78	47.6 ± 1.7	1052 ± 5.2
15 wt % BSA	1.64 ± 1.20	43.8 ± 2.3	1067 ± 6.7
10 wt % BSA with 1 μg/mL SiQD	1.43 ± 0.82	47.4 ± 2.3	1052 ± 5.3
10 wt % BSA with 5 μg/mL SiQD	1.44 ± 0.64	47.2 ± 1.8	1052 ± 5.2
10 wt % BSA with 10 μg/mL SiQD	1.45 ± 0.78	46.7 ± 1.7	1053 ± 2.1
10 wt % BSA with 25 μg/mL SiQD	1.46 ± 0.83	45.6 ± 1.2	1053 ± 5.3
10 wt % BSA with 50 μg/mL SiQD	1.46 ± 0.73	44.8 ± 1.5	1054 ± 5.4
10 wt % BSA with 100 μg/mL SiQD	1.48 ± 0.86	43.2 ± 1.4	1055 ± 5.4

### Microbubble Preparation

**Figure 2:**
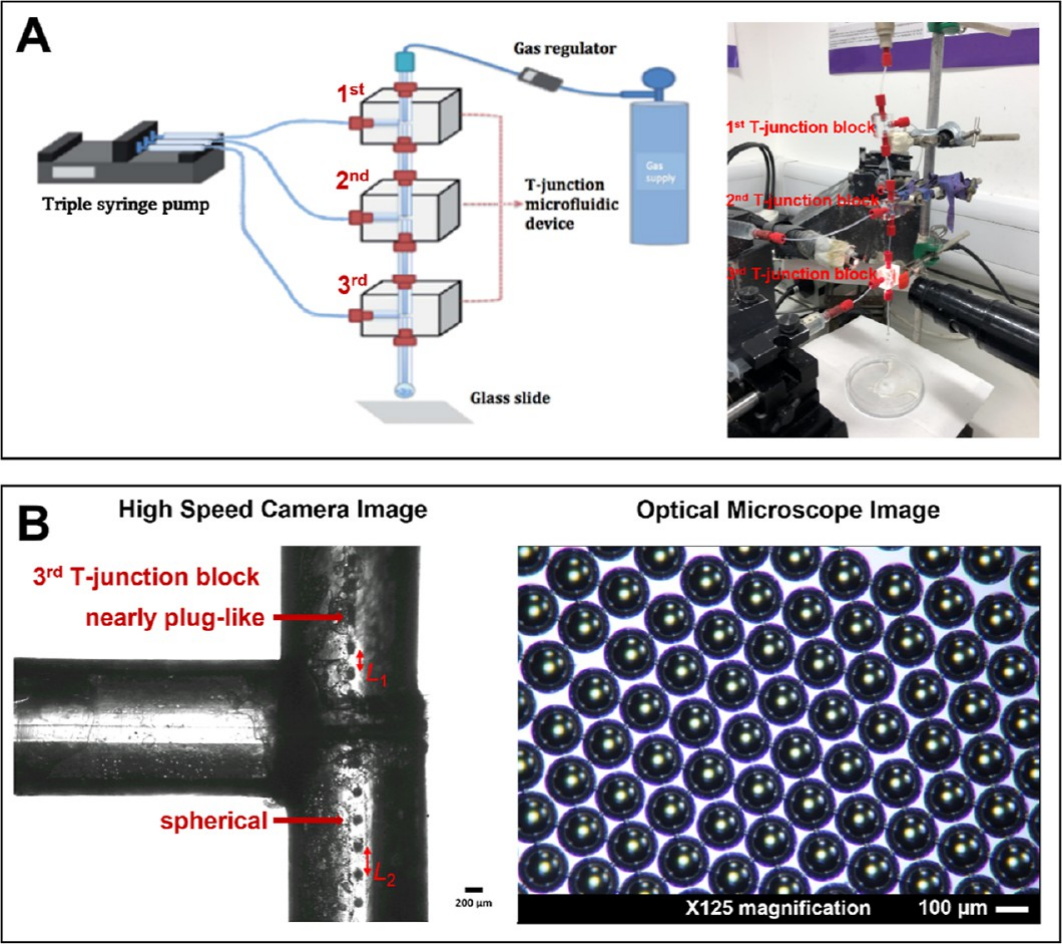
(A) Schematic diagram illustrating the triple T-junction microfluidic device setup of BSA solutions to prepare microbubbles. (B) High-speed camera and optical microscope images of microbubbles generated in the triple T-junction (with 200 μm capillaries) microfluidic device at a given gas pressure of 80 kPa with solution flow rate of 400 μL min^–1^; the diameter of microbubbles is 166 ± 11 μm.

Microbubbles were prepared using the T-junction microfluidic setup presented in Figure 2A at room temperature. Details of device components are given in ref (25). In summary, the device comprised two Teflon fluorinated ethylene polypropylene (FEP) capillary tubes (outer diameter: 1.59 mm; inner diameter: 200 μm or 100 μm) incorporated perpendicular to each other in a polydimethylsiloxane (PDMS) block (100 × 100 × 10 mm) to be the inlet channels for the gas (nitrogen, dispersed phase) and solution (BSA, continuous phase) flows. The inner diameter of the Teflon FEP capillary tubes was fixed either at 200 or at 100 μm for various groups of experiments. A third Teflon FEP capillary tubing was incorporated into the PDMS T-junction block co-axially aligned with the gas inlet channel. The gap between the aligned capillaries where the confluence of the two phases occurs was kept constant at 200 μm. Subsequently, a second and a third identical T-junction blocks were inserted in series to the exit channels of the first and second T-junctions, respectively. Furthermore, the lengths of the outlet capillary and middle connection capillaries between each two T-junctions were approximately ∼50 mm, in order to achieve the same capillary hydraulic resistance and flow resistance.

Gas was supplied by means of tubing (6 mm) to the top of the inlet channel of the first T-junction. The tubing was linked to a gas regulator connected to a pressurized nitrogen tank to provide gas at a controllable working pressure. A digital manometer was fitted to this tubing to measure the in-line gas pressure.^26^ The continuous phase BSA solution was infused at a constant liquid flow rate to all three T-junction inlets with the help of syringe pumps (Harvard Apparatus Ltd., Edenbridge, UK) to allow non-pulsating liquid flow into the T-junction, and three individual syringe pumps were used and set to the same liquid flow rate to assure equal distribution to all solution inlet channels. This was regulating checked by calibration. Care was taken to secure the capillary channels to prevent leakage of gas or liquid using high grade HPLC (Figure 2A).

Comparative experiments were carried out to investigate the influence of the additional T-junction(s) in series on microbubble formation. First, microbubbles were collected on glass slides from a single T-junction to analyze the bubble size. Subsequently, the second and third T-junctions were connected to the exit channel of the primary T-junction, while keeping the continuous phase solution the same for all solution inlet channels. Then microbubbles were collected from the double and triple T-junction setup, respectively.

For each of the single, double, and triple T-junction geometries, microbubbles were obtained at various flow rates (200, 400, 600, and 800 μL min^–1^) under constant gas pressure (80 kPa). To determine the effect on microbubble stability by SiQD, number of T-junctions, capillary size, liquid flow rate, and BSA concentration, the average microbubble diameter produced for a given liquid flow rate, gas pressure, and number of T-junctions at different BSA concentrations was measured and then calculated over a period of time. 100 microbubbles were chosen for each sample; optical micrographs were taken every 5 min until the disappearance of all microbubbles or the drying of their BSA shell. All experiments were carried out under ambient room temperature (∼23 °C) and relative humidity (40%); different environmental conditions were not explored.

### Microbubble Characterization

Microbubbles were immediately observed upon collection on a glass slide, using an optical microscope (Nikon Eclipse ME 600) fixed to a camera (JVC KY-F55B), with magnifications of 125×, 250×, 500×, 1000×, and 2000×. 100 microbubbles were selected at random for each sample to estimate the diameter and dissolution stability of microbubbles over a fixed collection area of 1.5 mm^2^. The mean diameter of microbubbles was obtained using ImageJ (National Institutes of Health, MD, USA, version 1.46r). In addition, fluorescence microscope (EVOS FL) was used to verify the presence of the SiQD layer on the microbubble shell. A Phantom V7.3 high-speed camera (Vision Research Ltd. Bedford, UK) with a recording time of 1.2 s and a resolution of 800 × 600 pixels at 4800 fps was used to obtain real-time video images of microbubble formation.

## Results and Discussion

### Effect of Additional T-Junctions on Microbubble Production

Comparative experiments were carried out to investigate the influence of connecting T-junctions in series on microbubble formation. High-speed camera snapshots were used to analyze how the additional T-junctions impact on the morphology of microbubbles within the capillary channels. The triple T-junction (with 200 μm capillary channels) was used at a constant gas pressure of 80 kPa, with a 15 wt % BSA solution infused at 400 μL min^–1^. Figure 2B shows how the shape of the microbubbles within the channels changed from plug-like elongated bubbles to spherical after they passed through the 3rd T-junction, resulting in a smaller microbubble diameter upon collection. Analysis of the high-speed camera images revealed that with the additional T-junctions, the distance between the microbubbles increased upon passing through the extra T-junction; the distance between the microbubbles prior to the passing of the 3rd T-junction was *L*_1_ = 240.4 ± 2.3 μm, which increased to *L*_2_ = 373.2 ± 2.8 μm after the 3rd T-junction. A similar trend was observed for higher liquid flow rates up to 800 μL min^–1^ at a constant gas pressure of 80 kPa. Introducing an extra solution flow through the addition of a T-junction increases the velocity of the microbubble flow in the outlet channel; accordingly, the microbubbles were pushed away from one another by the liquid phase, causing the increased distance between adjacent microbubbles. This mechanism can be exploited for reducing microbubble coalescence within the micro-channels before bubble collection.

### Effect of Working Pressure, Liquid Flow Rate, Solution Concentration, and Capillary Channel Size on Microbubble Size

To determine the minimum gas pressure that enables microbubble production for the triple T-junction assembly with 100 μm capillary channel size, the working pressure was gradually increased for a given maximum solution flow rate (800 μL min^–1^) until the stable microbubble formation regime was achieved; this minimum gas pressure was found to be 80 kPa, which was subsequently kept constant as the working gas pressure for all single, double, and triple T-junction experiments. The smallest microbubbles obtained in this work were 22.8 ± 1.4 μm in size, generated using a triple T-junction at a minimum gas pressure of 80 kPa coupled to the maximum liquid flow rate at 800 μL min^–1^, with 15 wt % BSA solution and a micro-capillary of channel diameter of 100 μm. 80 kPa gas pressure is the minimum gas pressure that allows continuous microbubble generation of this diameter, if we have three T-junctions with 100 μm capillaries into which the BSA solution is pumped at 800 μL min^–1^.

It has to be emphasized that this work was not targeted to making microbubbles of a size with the eventual goal of meeting the biomedical application requirement of <5 μm diameter microbubbles. This paper elucidates the novel science and engineering of connecting multiple T-junctions.

**Figure 3:**
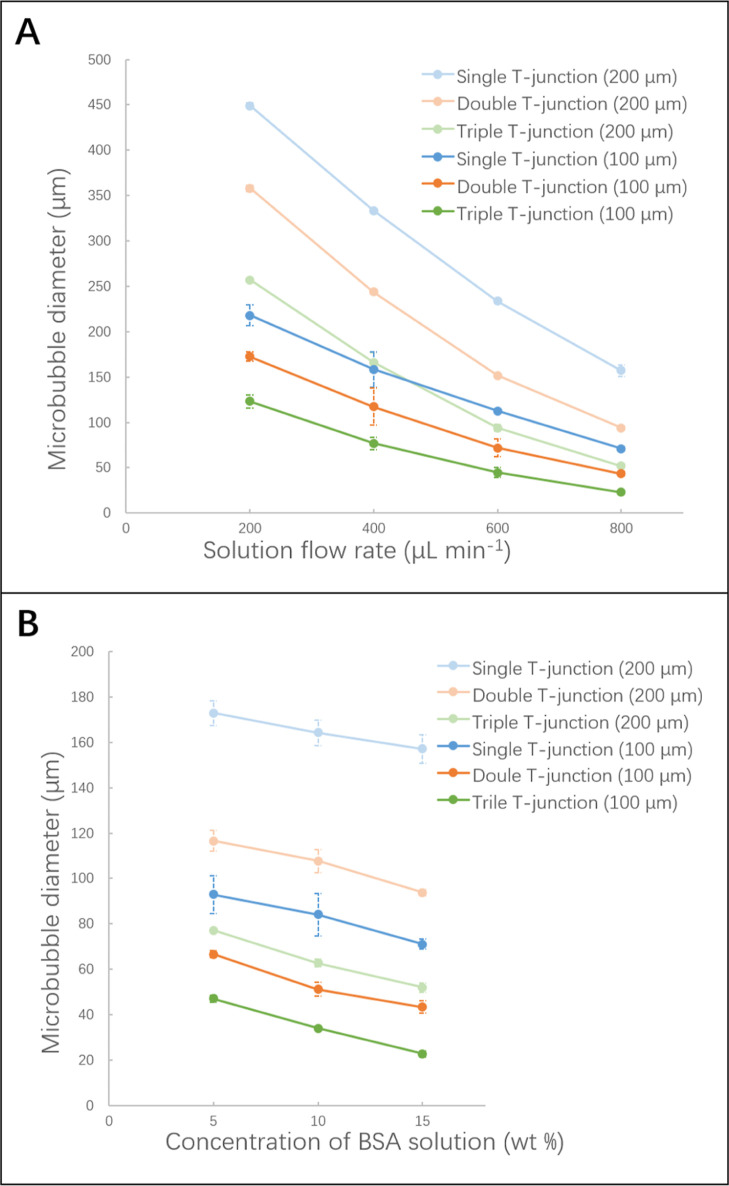
(A) Relationship between microbubble size and solution flow rate (15 wt % BSA); here microbubbles were generated using various T-junctions working at a constant pressure of 80 kPa. (B) Relationship between microbubble size and concentration of BSA solution with flow rate set at 800 μL min^–1^; here microbubbles were produced using various numbers of T-junctions working at a constant pressure of 80 kPa.

As shown in Figure 3A, the largest microbubble size was obtained from the single T-junction with a 200 μm channel at the lowest liquid flow rate, which is 200 μL min^–1^, and under a constant gas pressure of 80 kPa, whereas the smallest size of microbubbles was obtained with the use of the triple T-junction with an inner capillary diameter of 100 μm and the highest solution flow rate (800 μL min^–1^) and at the same constant working pressure. Subjected to the lowest liquid flow rate of 200 μL min^–1^, and for the same capillary size (with either 100 or 200 μm capillaries), microbubbles generated with the triple T-junction were smaller and more uniform as compared with those produced by double and single T-junctions. Specifically, the diameter of microbubbles obtained in the triple T-junction was smaller at 123.2 ± 7.1 μm for 100 μm capillary and 257.1 ± 1.5 μm for 200 μm capillary, compared to those produced using double T-junction at 172.7 ± 5.0 μm for 100 μm capillary size and 358.0 ± 3.3 μm for 200 μm capillary and those from single T-junction at 218.0 ± 11.6 μm in size for 100 μm capillary and 449.3 ± 2.5 μm for 200 μm capillary (Figure 3A). Moreover, at the highest solution flow rate of 800 μL min^–1^, microbubbles of lowest sizes of 22.8 ± 1.4 μm were obtained for 100 μm capillary and 51.9 ± 2.0 μm for 200 μm capillary in triple T-junction, compared to those from double T-junction at 43.3 ± 2.6 μm for 100 μm capillary and 93.7 ± 1.3 μm for 200 μm capillary, and those from single T-junction at 71.0 ± 2.3 μm for 100 μm capillary and 157.0 ± 6.2 μm for 200 μm capillary. When keeping liquid flow rate and working pressure constant, the triple T-junction geometry generated monodispersed microbubbles with smaller diameters at all times. Moreover, the microbubbles produced through higher solution flow rates were smaller as compared with the ones through the lower solution flow rate applied for all of the single, double, and triple T-junction microfluidic setups (Figure 3A), in agreement with the literature that reports that microbubble size strongly depends on liquid flow rate under a constant gas pressure.^24^

Figure 3B shows a gradual reduction in microbubble diameter with increasing BSA concentration from 5 to 15 wt %; this parameter is particularly significant for the triple T-junction setup. Microbubble size depends on the balance of capillary force, Laplace pressure, surface tension, and liquid shear stress force.^27^ The solution viscosity and surface tension increase with the BSA concentration, which can reduce the size of microbubbles due to the increase in cross-flow shear force over the capillary force at the channel junction. Beyond 15 wt % BSA, the capillaries are frequently clogged; hence, the 15 wt % was selected as an upper limit of the solution concentration, which reliably ensured freedom from clogging.

**Figure 4:**
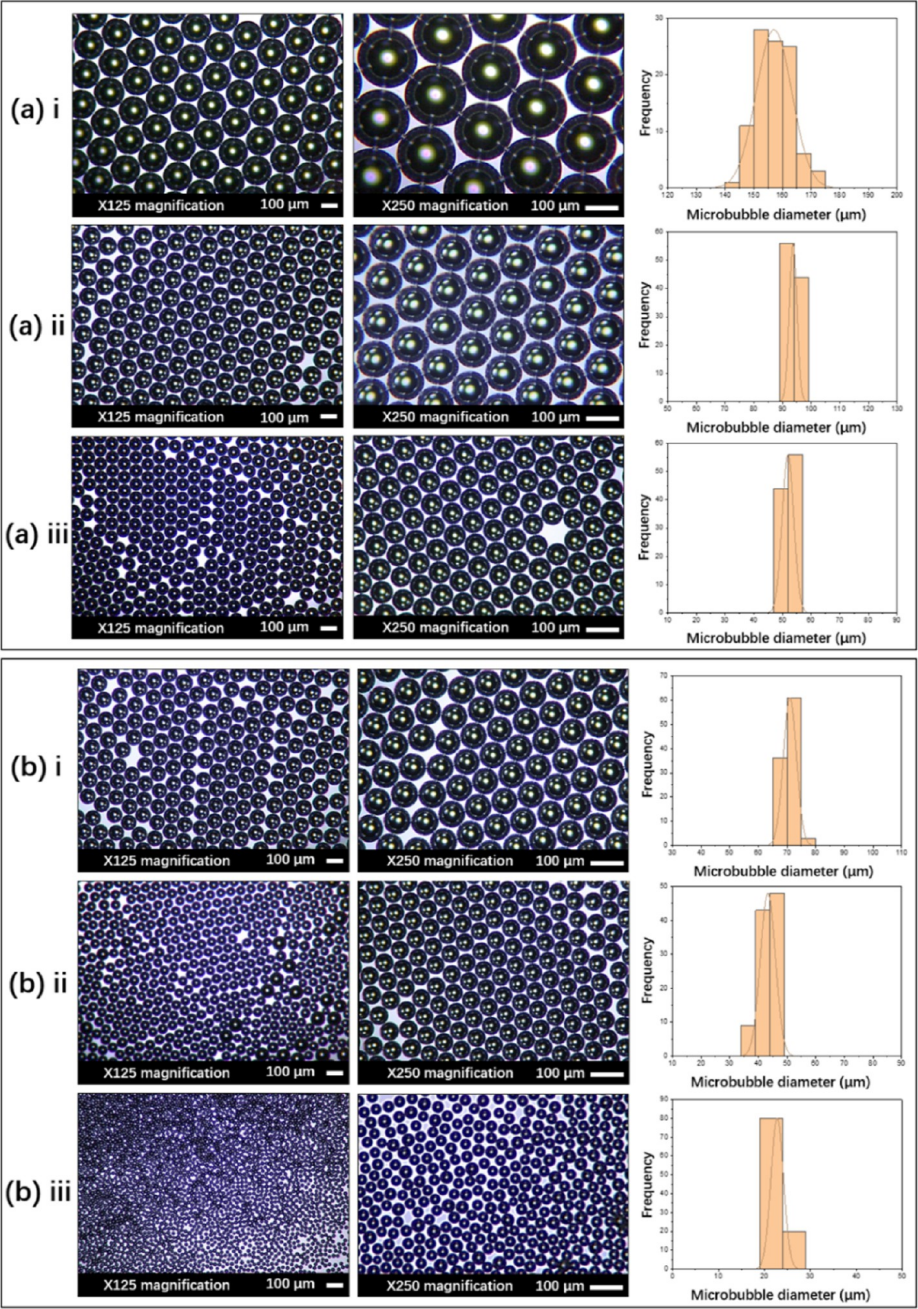
Optical micrographs and corresponding size distribution diagrams of microbubbles generated using 15 wt % BSA solution at a constant liquid flow rate of 800 μL min^–1^ and a gas pressure of 80 kPa with a capillary size of (a) 200 μm and (b) 100 μm, in the (i) single, (ii) double, and (iii) triple T-junction microfluidic setups. The microbubble diameter for each case is (a) (i) 157 ± 6 μm; (ii) 93.7 ± 1.3 μm; (iii) 51.9 ± 2.0 μm and (b) (i) 71.0 ± 2.3 μm; (ii) 43.3 ± 2.6 μm; (iii) 22.8 ± 1.4 μm.

Under constant flow rate, BSA concentration, and gas pressure, the 100 μm capillary channel provides monodisperse microbubbles with a smaller diameter at all times (Figures 3 and 4). This is consistent with the literature; the internal diameter of the capillary channel has a dominant influence on microbubble size.^24^ Nonetheless, one of the main challenges with T-junction microfluidic devices is to produce microbubbles suitable for biomedical applications (microbubble size <10 μm);^18^ to do this, it is necessary to use capillary channels with very small diameters, which are easily clogged by residue solution; pumping a viscous liquid through such small micro-channels is unfeasible, thereby limiting the variety of solutions and their concentrations that can be used. Although microbubble stability and not size was the focus of investigation in this work, it is interesting to note that further reduction of microbubble diameter would be possible by connecting more T-junctions in series, although this is not the focus of this work.

### Computational Modeling of Microbubbles Fabricated Based on Experimental Conditions

**Figure 5:**
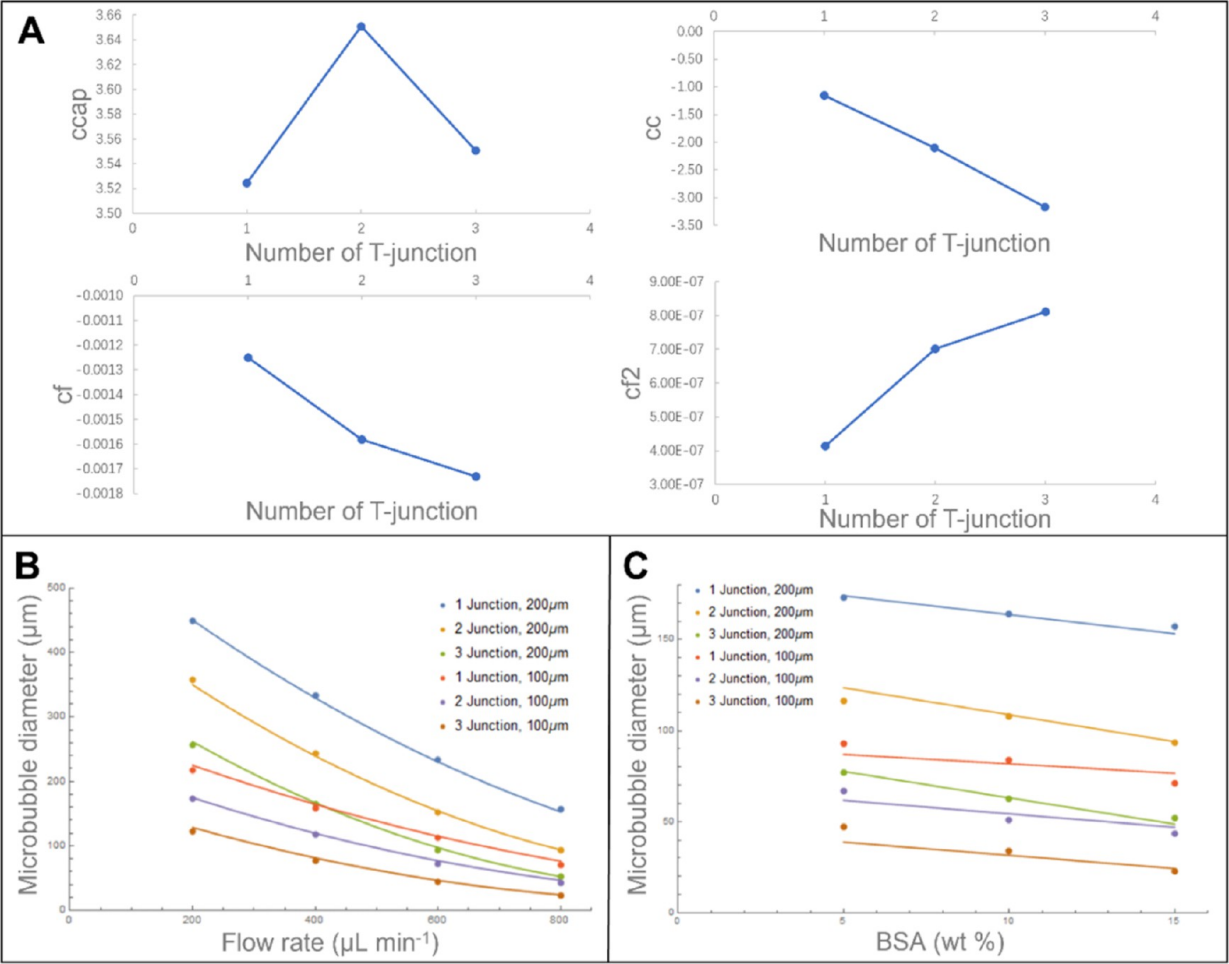
(A) Variation of the fitting parameters in eq 1 with the number of T-junctions *n*T. (B) Variation of microbubble diameter with liquid injection flow rate. The points are the experimental results, and the curves are from a fit of eq 3. (C) Variation of microbubble diameter with BSA concentration. The points are the experimental results, and the curves are from a fit of eq 3.

A data-fitting exercise has been applied: the aim is to mathematically describe the approach taken and to summarize the best results obtained in this work (for this reason, the values of the fitting parameters are omitted). Overall, 36 different experimental conditions were used, varying the number of junctions, concentration, and liquid flow rate, and in each case, the mean and standard deviation of the bubbles produced was established. For initial analysis, from looking at a selection of plots of microbubble diameter against one of the control variables, namely, the number of T-junctions *n*, the capillary diameter *d*, the liquid input flow rate *f*, and the BSA concentration *c*, it appeared that for each value of *n*, the bubble size varied smoothly with *f* and *c*: some experimentation suggested that a linear dependence on *d* and *c* and a quadratic dependence on *f* provided relatively good fits. As is conventional, the statistics of the fit, especially the *p*-values, were used to assess the significance of the various terms in the expressions being used. The first fitting carried out treated each number of T-junctions separately and used an expression of the form
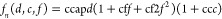
1with calibration parameters varied to optimize the fit, namely, the diameter of capillary ccap, the first order of liquid flow rate cf, the second order of liquid flow rate cf2, and the concentration of BSA solution cc. With this form of expression, the 12 results for one T-junction could be fitted with root mean square error of 3.93 μm in the diameters. For two and three T-junctions, the root mean square errors were 3.48 and 3.35 μm, respectively. When the variation of the fitting parameters was considered as a function of the number of T-junctions, as shown in Figure 5A, there appeared to be an almost linear variation (with the exception of ccap, but the variation in that parameter was relatively small).

For overall fitting, with the guidance from the initial analysis, attempts were made to fit the entire data set. It had been hoped that an iterative model might have been applicable, with each junction modifying the output of the previous one in a consistent manner, leading to
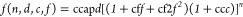
2but the resulting fits were poor. Instead, a more general form was adopted, with the microbubble diameter being expressed as
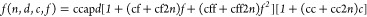
3

This allowed a good fit across the whole data set, with a root mean square error of 4.06 μm. The quality of the fit is demonstrated by Figure 5B,C.

It should be noted that the number of parameters in the fit (7) is relatively large compared with the number of data points (36). It is important to check that there is no overfitting, and here, we used additional data to see whether extrapolation beyond the fitting range is successful. Previous research work^28^ did not extend to the pressure used in this work, but little error is likely to arise from using their linear fits to extrapolate from their maximum pressure of 70 and 75 kPa to the 80 kPa applied here. If we do this, we can also explore how eq 3 may be used to extrapolate from the minimum liquid injection rate of 200 μL min^–1^ to the 100 μL min^–1^ applied in previous work.^28^ The agreement, as shown in Table 2, is satisfactory. This suggests that eq 3 provides a good representation of the behavior, at a pressure of 80 kPa, of up to three junctions under a variety of liquid injection flow rates.

**Table 2:** Comparison of the Prediction of eq 3 with Experimental Results Which Were Not Used in the Fitting Process

T-junction series	capillary diameter (μm)	pressure (kPa)	liquid Flow rate (μL min^–1^)	BSA concentration (wt %)	experimental microbubble diameter (μm)	predicted microbubble diameter (μm)
2	100	80	100	15	221	207
2	100	80	200	15	179	175
1	100	80	100	15	267	259
1	100	80	200	15	225	225

**Figure 6:**
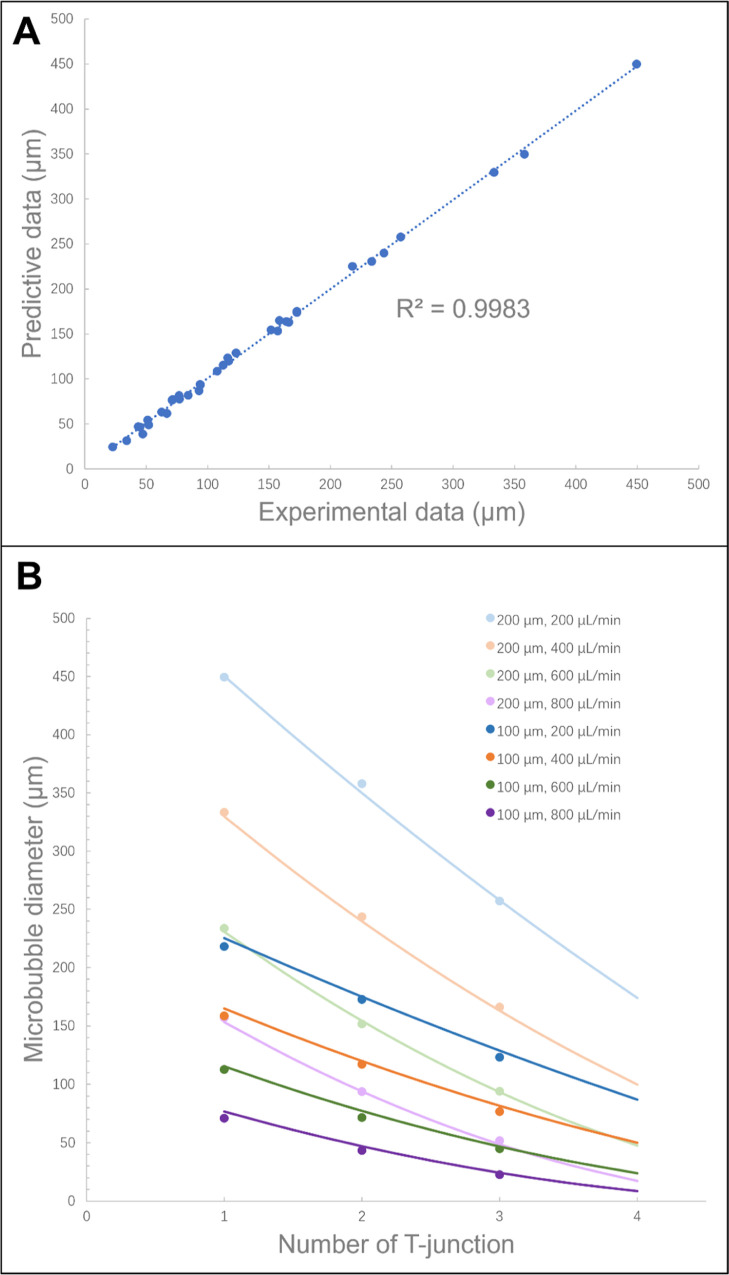
(A) Predicted modeling best-fit line agreed with experimental data. (B) Variation of microbubble diameter with the number of T-junction. The points are the experimental results, and the curves are from a fit of eq 3.

In order to provide a computational model that can predict the diameter of microbubbles fabricated by incorporating the number of T-junctions in this model as one of the variables, it was illustrated that the diameter of the monodisperse microbubbles generated can be tailored using multiple T-junctions, while the operating parameters were kept constant. For the given liquid flow rate and BSA concentration exploited in this work with a capillary size of either 100 or 200 μm at a constant working pressure of 80 kPa, the number of T-junctions required for generating microbubbles of desired diameter can be predicted from this computational model. Figure 6B demonstrates those predictions and suggests that a 100 μm diameter capillary tubing be employed at a given liquid flow rate of 800 μL min^–1^ with 15 wt % BSA solution under the constant working pressure of 80 kPa.

### Stability Analysis of Microbubbles Produced Using Various Solution Concentrations and T-Junction Numbers

**Figure 7:**
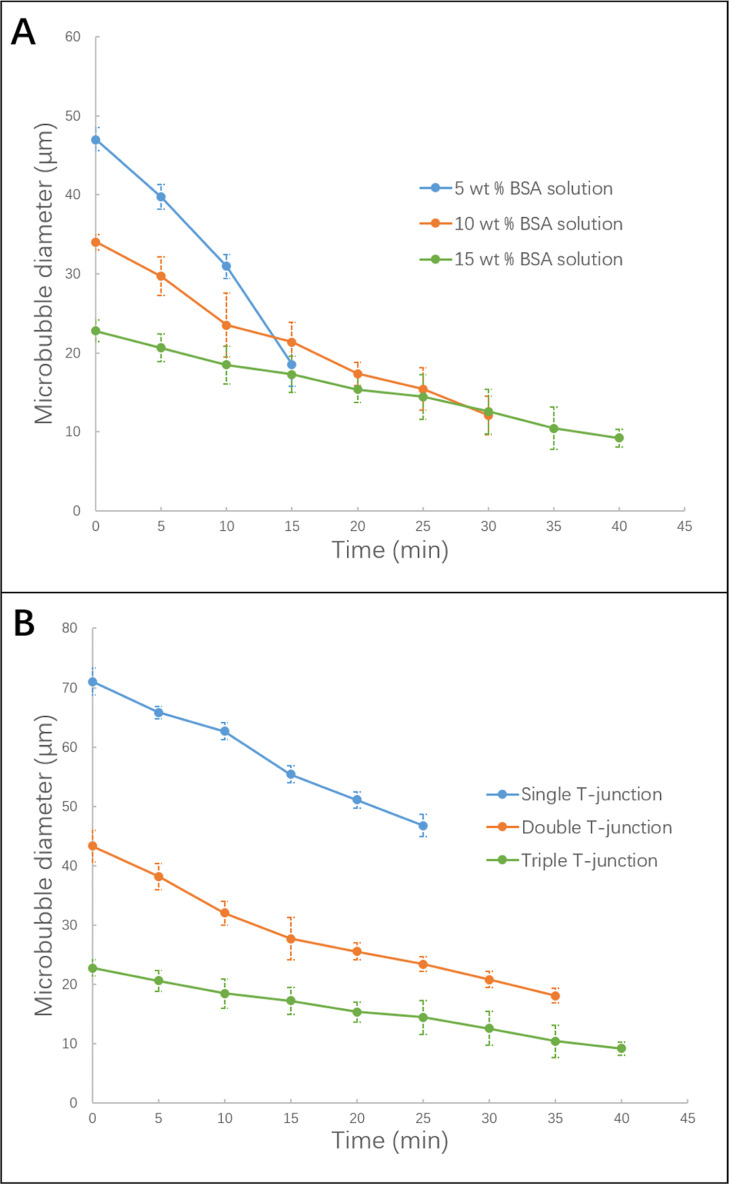
(A) Lifetime of microbubbles (stability) for triple T-junction with 100 μm capillary at the same liquid flow rate (800 μL min^–1^) and gas pressure (80 kPa). (B) Lifetime of microbubbles using 15 wt % BSA solution and different number of T-junctions (100 μm capillary) at constant liquid flow rate (800 μL min^–1^) and gas pressure (80 kPa).

**Figure 8:**
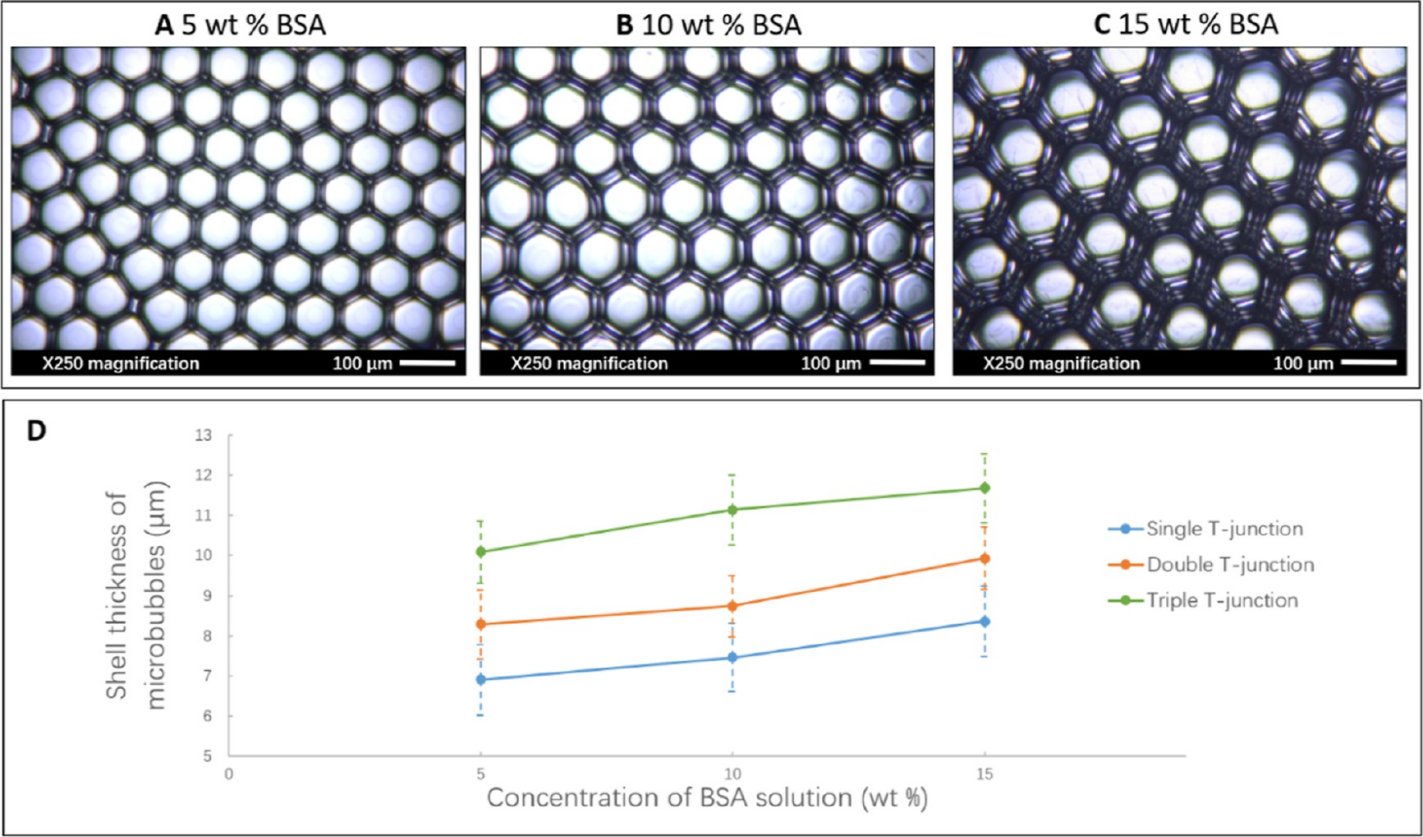
(A–C) Optical micrographs of dried BSA microbubbles made by triple T-junction showing that the shell thickness increased with increasing BSA concentration. (D) Shell thickness of microbubbles increased with increasing concentration of BSA solution for all T-junctions (100 μm capillary size) at constant liquid flow rate (800 μL min^–1^) and gas pressure (80 kPa).

Figure 7A shows the stability analysis of the microbubbles generated using various concentrations of BSA solution. Note that while the single T-junction microbubble lifetime measurement is terminated at the point when the microbubbles burst, the double and triple T-junction bubble lifetime analysis is terminated at the time point when the bubbles become dry, as they do not appear to burst after collection on the glass slide. According to optical micrographs, all stability measurements were terminated at the disappearance of all microbubbles or the drying of their BSA shell. This result shows that the increasing concentration of the BSA solution (5, 10, and 15 wt %) has a linear effect on the stability of microbubbles in addition to reducing bubble size, as shown earlier in Figure 5C. According to Figure 7A, mean microbubble diameter decreased with holding time post-collection and the most stable microbubbles were the smallest in diameter at 22.8 ± 1.4 μm, which showed a stability of up to 40 min subsequent to collection on a glass slide at 40 min, when the bubbles dry to generate a bubble skeleton (scaffold). These microbubbles are produced from 15 wt % BSA solution (highest concentration) through the triple T-junction with the 100 μm capillaries at the maximum liquid flow rate of 800 μL min^–1^ under a minimum gas pressure of 80 kPa. Conversely, the microbubbles produced under the same condition using 5 wt % BSA solution (lowest concentration) were observed to exhibit the lowest stability (stable for 15.0 ± 0.8 min), with a mean diameter of 47.0 ± 1.4 μm. This suggests that microbubbles with low BSA concentration produce microbubble shell with lower resistance for gas diffusion from the microbubble core to the surrounding, leading to quicker dissolution as compared to microbubbles produced at a high BSA concentration. Figure 7B shows the significant effect of T-junction numbers on microbubble stability. The microbubbles obtained through the use of the triple T-junction were more stable under the same conditions (for a given liquid flow rate, gas pressure, solution concentration, and capillary size) compared to those generated through double and single T-junctions. Specifically, microbubbles produced by a single T-junction showed a stability period of 25.0 ± 1.3 min, in contrast to those collected from the double and triple T-junctions, which were stable for 35.0 ± 1.8 and 40.0 ± 2.0 min, respectively. In addition, Figure 8 shows that the dried microbubble shell thickness increases with increasing BSA concentration.

Microbubbles shrink in size with time due to gas dissolution to the surroundings. Figure 7 shows that the stability of the microbubbles improved with a higher concentration of BSA solution and a higher number of T-junctions. The improved stability is attributed to the increase in microbubble shell thickness, as shown in Figure 8. Specifically, the elasticity of the microbubble interface increases with the thickness of the shell; hence, thicker shelled bubbles avoid shrinkage due to disproportionation.^29^ As mentioned earlier, the higher the concentration of the BSA solution, the greater the shell thickness (Figure 8A–C), and consequently, the stronger the shell elasticity. Furthermore, Figure 8D shows that increasing the number of T-junctions in series also directly increases the shell thickness of microbubbles produced; the triple T-junction microbubbles were thicker than those from double and single T-junctions at the same BSA concentrations. This is attributed to the additional BSA solution coating through the liquid phase infused at the added T-junction. Through this means, the serial T-junction setup developed here has the potential to generate multi-layered microbubbles. The shell thickness increases, providing a greater shell thickness that prevents the rapid diffusion of gas, promoting stabilization of the microbubbles.

### Mathematical Modeling of Microbubble Lifetime Evolution in Air

This section was prompted by the present research works’ observations, of the lifetimes of encapsulated microbubbles generated by a microfluidic process. Although the evolution of bubble sizes has been studied before, both for simple bubbles^30^ and for encapsulated bubbles,^31^ previous work^32−34^ has generally addressed bubbles immersed in a liquid. Here, we extended the work to the case of free encapsulated bubbles, that is, bubbles in a gaseous environment.

Theoretically: we consider a spherical bubble with a permeable shell with inner radius *a* and outer radius *b*. We shall also use the notations *a*_–_ and *a*_+_ to denote the position next to the shell in the gas-filled core and the position in the shell closest to the core; we shall define *b*_–_ and *b*_+_ in a similar way. For the moment, we will suppress the time dependence of *a* and *b* in the mathematical expressions. We will assume that gas diffusion through the shell is sufficiently slow compared with mixing processes in the gas and that the gas concentration may be taken as constant (albeit with different values) both inside and outside the shell. The concentration of the gas *c*_g_, which is convenient to measure in moles m^–3^, is a function of radial position *r* and time *t* denoted by *c*_g_(*r*,*t*), and its diffusion through the shell if we neglect transients may be described by
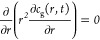
4which may be solved to give
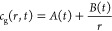
5where *A*(*t*) and *B*(*t*) depend on the concentrations at the surfaces of the shell. If the shell is thin (*b*_–*a*_ = δ ≪ *b*) then the outward flux of gas will be
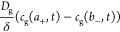
6*D*_g_ being the diffusion coefficient of the gas in the shell. We may equate the total gas flow to the rate of change of the number of moles of gas inside the spherical bubble, *m*_g_, which is
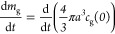
7

The concentration just inside the surface of the shell is related to that just outside by the Ostwald coefficient *L*_g_ through
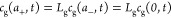
8and similarly at the outer surface at *r* = *b*.

For *m*_g_ moles of a perfect gas at temperature *T* in volume *V*, we have
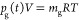
9
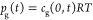
10where *R* is the universal gas constant. In the bubble, contained by an inner and an outer surface with surface tension γ
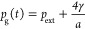
11where *p*_ext_ is the external ambient pressure.

Putting this together, we find
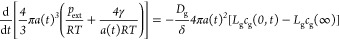
12where we have replaced  with *c*_g_(*∞*) and included the time dependance of *a* explicitly. Thus
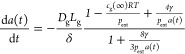
13

We can now follow Katiyar and Sarkar^35^ by introducing the elasticity of the shell. Thus, we modify the surface tension as
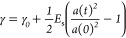
14where γ_0_ is the surface tension and *E*_s_ is the shell elasticity, which may be written as
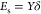
15where *Y* is Young’s modulus for the shell. We assume that the shell will buckle when under compression, and so we take the limit of stability as the time at which γ passes through zero. This leads to a revised differential equation
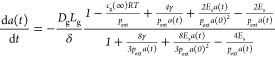
16

So far, we have considered diffusion in only one direction, with gas diffusing out of the microbubble. In reality, we start with a microbubble filled with nitrogen, which is surrounded by air, which we may approximate as a mixture of nitrogen and oxygen. There is, therefore, a concentration gradient down which oxygen will diffuse into the microbubble. This leads, in the case in which we consider only surface tension, to a set of three coupled differential equations that have to be solved simultaneously (we have replaced the subscript g with specific labels for nitrogen N and oxygen O)
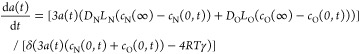
17
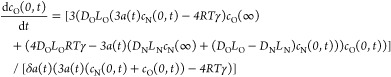
18
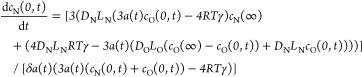
19Introducing elasticity to the shell membrane leads to
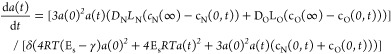
20
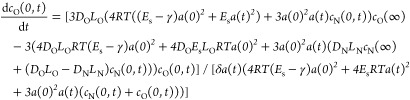
21
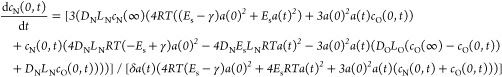
22

For initial numerical experiments, we have nitrogen in the microbubbles, which is 78% of the composition of air, so the exterior pressure of nitrogen is 0.78 times the total external pressure. We will assume that the gases diffuse through the shell by dissolving in the residual water from the BSA solution. The Ostwald coefficient for nitrogen in water is given by *L*_N_ = 6.4 × 10^–6^*RT* mol m^–3^.^36^ We take the diffusion constant to be one-tenth of the diffusion constant for nitrogen in water, which is *D*_N_ = 1.88 × 10^–9^ m^2^ s^–1^.^37^ We estimate the elastic properties of BSA by interpolating from published results, which give a tensile Young’s modulus of 7.5 kPa for 5% and 16.5 kPa for 9% BSA.^38^ We therefore estimate 18 kPa for 10% and 28 kPa for 15% BSA. The surface tension of 15% BSA is measured at 51.3 mN m^–1^, of 10% BSA at 47.6 mN m^–1^, and of 5% BSA at 43.8 mN m^–1^.

**Figure 9:**
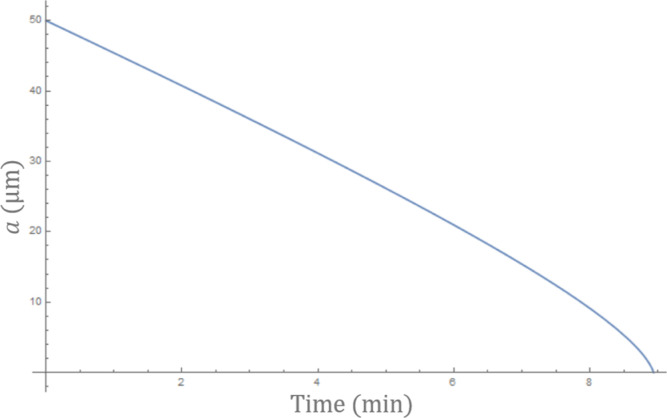
Variation in size of an elastic shell (*a*) as a result of gas diffusion as predicted by the model.

If we assume an initial radius of *a* = 50 μm and shell thickness of 10 μm, we can solve the differential eq 16 numerically. The result is shown in Figure 9, and the decay time for the bubbles is of the order of 10 min, which is of the same order of magnitude as the observed bubble lifetime. In view of the uncertainty in some of the parameters of the model (notably bubble wall thickness and the diffusion coefficient of nitrogen through the wall), this suggests that the model captures the important features of bubble decay. There are several ways in which the model can be improved in future work. First, the microbubble reduces to an internal radius of zero. As we have considered only the diffusion of nitrogen, this is inevitable; in reality, there will be diffusion of oxygen inwards. Second, we have used equations that apply in the case of a thin shell, and this may not be sufficiently accurate for the relatively thick shell microbubbles that were produced by the experiments carried out in this work.

### Effects of Silicon Quantum Nano-Dots on the Stability of Microbubbles

**Figure 10:**
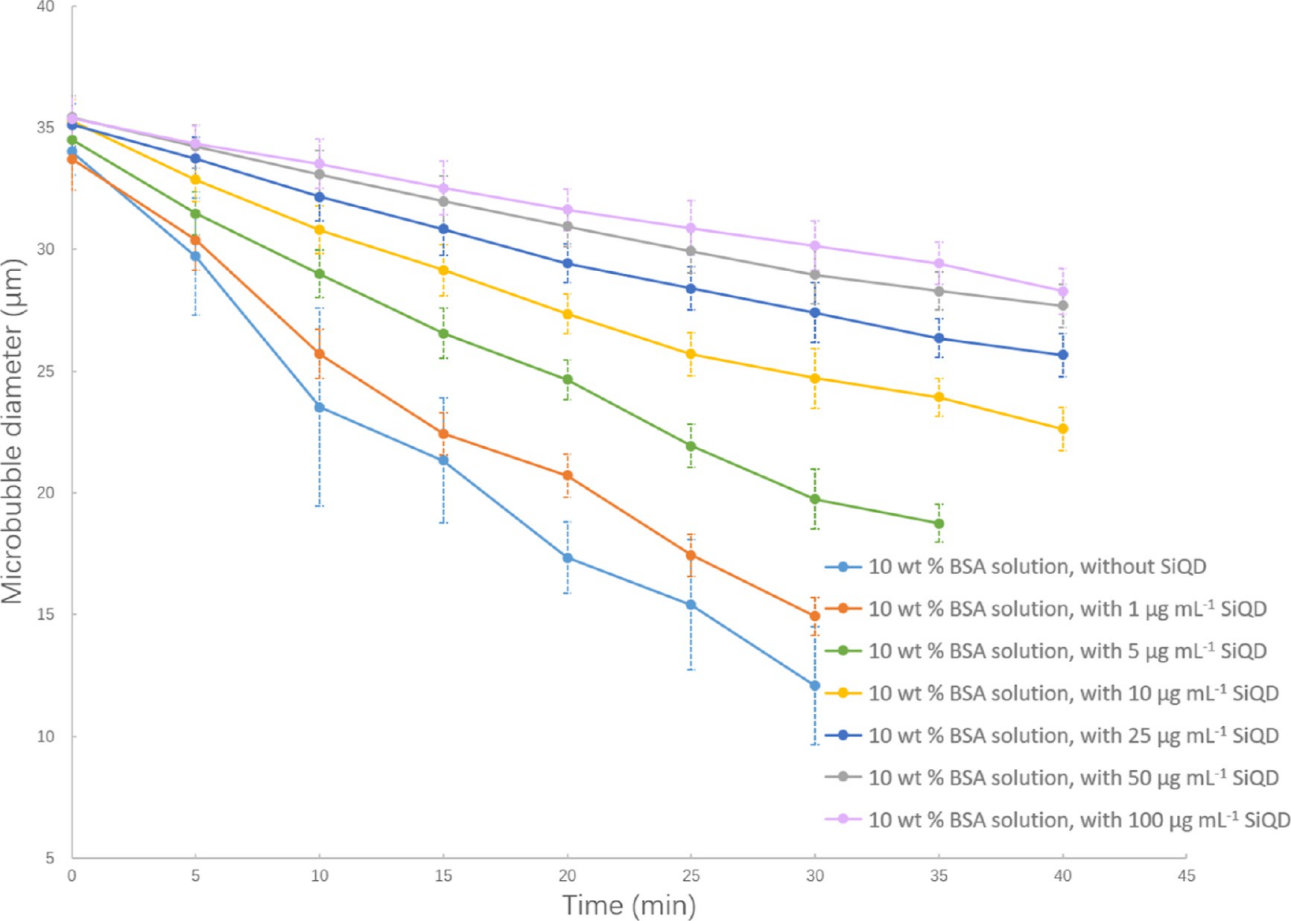
Graph illustrating the lifetime of microbubbles (stability) of microbubbles generated using the triple T-junction (100 μm capillary) at the same flow rate (800 μL min^–1^) and gas pressure (80 kPa).

**Figure 11:**
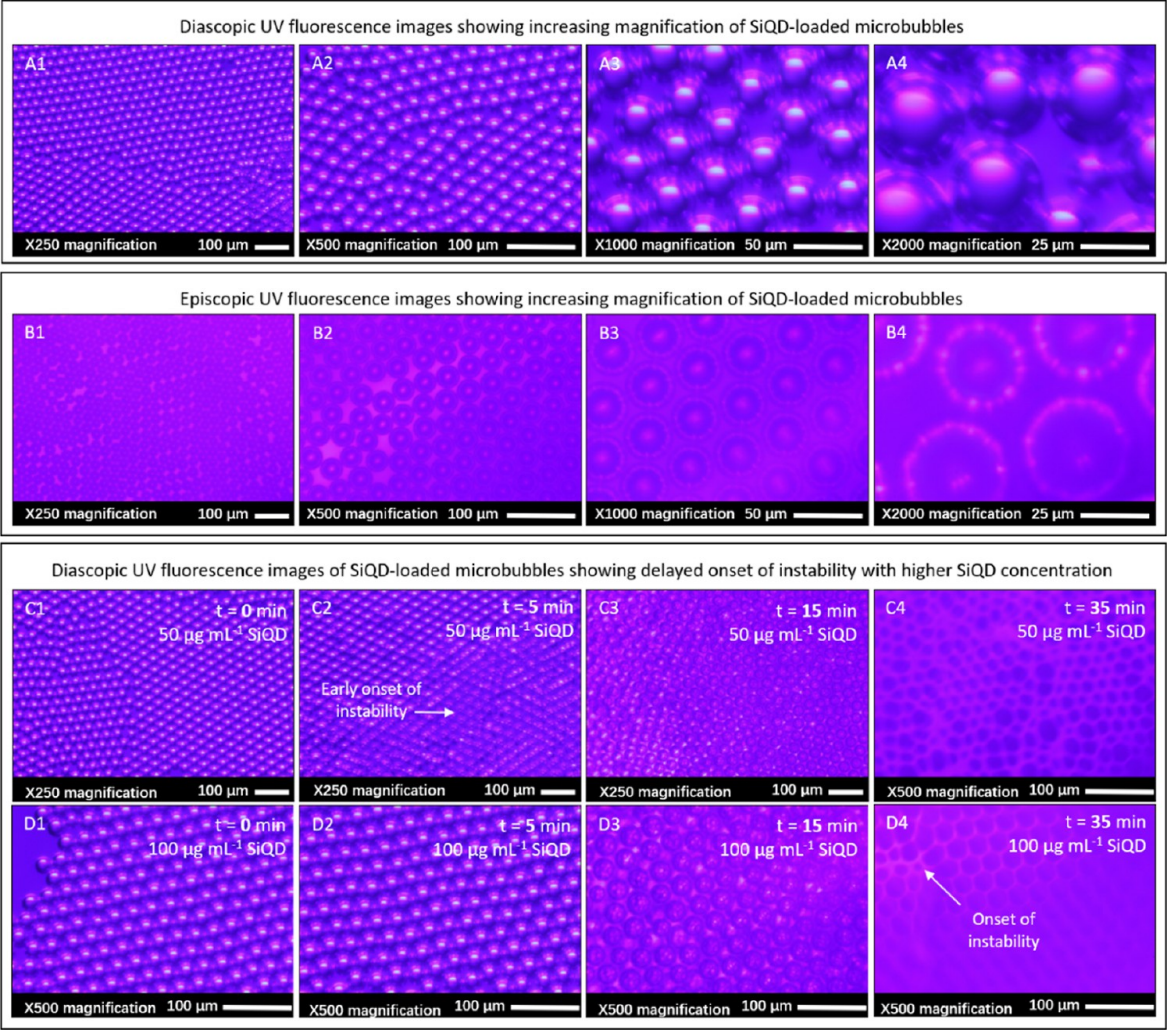
(A1–A4) Diascopic and (B1–B4) episcopic UV fluorescence images showing increasing magnification of 100 μg mL^–1^ SiQD-loaded microbubbles. (C,D) Diascopic UV fluorescence images at varying time points showing stability of (C1–C4) 50 μg mL^–1^ SiQD-loaded microbubbles and D1-4 100 μg mL^–1^ SiQD-loaded microbubbles. Delayed onset of instability was observed with higher SiQD concentration. Microbubbles were produced from 10 wt % BSA using a triple T-junction (100 μm) under constant flow rate (800 μL min^–1^) and gas pressure (80 kPa).

Figure 10 shows the stability analysis of the microbubbles fabricated through the addition of various concentrations of SiQD. The binding of SiQD to the microbubble surface has been verified under fluorescence microscopy (Figure 11). The bright shell of the microbubbles (Figure 11) indicates the presence of SiQD. The microbubbles generated from BSA solutions without SiQD shrank more significantly in size over time than the SiQD-loaded BSA microbubbles (Figure 10). Also, it has been experimentally observed that (Figure 10) the BSA microbubbles generated with 1 μg mL^–1^ and without SiQD were non-existent after 30.0 ± 1.5 min (lifetime period). The BSA microbubbles produced with 5 μg mL^–1^ SiQD retained morphology until their BSA shell dried after 35.0 ± 1.8 min. The BSA microbubbles produced with 10, 25, 50 and 100 μg mL^–1^ SiQD retained morphology until their BSA shell dried at 40.0 ± 2.0 min. There is no significant effect of SiQD on the microbubble dissolution when a low SiQD concentration was used (1–5 μg mL^–1^); nevertheless, the onset of instability of microbubbles was delayed, compared with BSA microbubbles without SiQD. A greater stability was achieved by the presence of a higher SiQD concentration (10–100 μg mL^–1^), which strongly prevented microbubble dissolution (Figure 11 panel C,D).

Liquid flow rates ranging from 200 to 800 μL min^–1^ were used to generate microbubbles using single, double, and triple T-junctions. The generation of microbubbles in T-junction microfluidics is governed by the pressure balance between the dispersed and the continuous phase at the junction, which in turn is controlled by the applied gas working pressure and the liquid flow rate.^39^ For a solution of a given concentration (constant viscosity and surface tension) and gas working pressure, monodisperse microbubble formation occurs in a specific range of the liquid flow rates, with the smallest microbubble produced at the maximum solution flow rate and vice versa.^40^ If the liquid flow rate is increased above a certain threshold, liquid dripping or capillary leakage occurs as the liquid phase pushes the inlet gas stream in a backward direction due to the capillary force of the liquid phase; conversely, jetting occurs with the decrease in liquid flow rate below this threshold, during which the laminar flow of the gas and liquid phases in the outlet channel is disturbed by the high-pressure gas stream, resulting in polydisperse microbubbles.^28^

With regard to the scenario of balancing liquid and gas pressure, with the pressure kept constant at the junction, increasing the liquid flow rate beyond a pressure threshold will force the gas backward and halt microbubble formation. For the production of microbubbles, the gas pressure needs to be sufficient to penetrate the solution channel to commence microbubble formation.^26^ Hence, at low liquid flow rates, microbubbling model begins at lower gas pressures. In contrast, higher solution flow rates require higher working pressure to enable stable microbubble formation. Moreover, gas pressure diminishes at the passing of each T-junction; hence, the double and triple T-junctions required higher minimum gas pressure to form microbubbles at the outlet channel than the single T-junction.

As presented above, the microbubble size reduction could be accomplished either by increasing the BSA solution concentration or by increasing the number of T-junction(s) applied in the system. The triple T-junction technique presented here has the potential to generate multi-layered microbubbles as the added junctions provide additional microbubble shell coatings. It was also shown that the microbubbles generated with a smaller size were more stable (Figure 7). This effect is most likely due to the fact that the microbubbles formed from a solution with the given viscosity have a constant surface tension that is responsible for cohesive forces between liquid molecules. In larger microbubbles, the gas–liquid interface is loosely packed, and gas dissolution is therefore more likely to occur.^29^ Conversely, smaller microbubbles with a more densely and tightly packed gas–liquid interface have been stable for a longer period of time. According to the Epstein and Plesset equation,^30^ the rate of gas dissolution and therefore the rate of change of microbubble size depend on factors such as surface tension and rate of gas diffusion through the liquid shell. Laplace pressure is inversely proportional to the diameter of the microbubbles, as the stability of the microbubbles exposed to atmospheric conditions is dominated by their diameter; the smaller size of the microbubbles has a lower gas exchange rate with the surroundings, thereby increasing stability.

While the mathematical modeling of the microbubble lifetime evolution in air agrees with the empirical results, there are several ways that the computational prediction can be improved in future work. First, the microbubble reduces to an internal radius of zero. As we have considered only the diffusion of nitrogen, this is inevitable: if the interior can contain only pure nitrogen, equilibrium with an external atmosphere containing 78% can be achieved only by completely emptying the interior. Second, we have used equations that apply in the case of a thin shell: this may not be sufficiently accurate for the bubbles that are produced by the experiments and that have relatively thick shells.

The attachment of SiQD to the microbubble surface is irreversible and stabilizes the microbubble surface. Thus, the formation of SiQD-loaded BSA microbubbles with enhanced stability is achieved through a balance between the propensity of the partially hydrophobic particles to adsorb onto BSA and their tendency to aggregate rather than disperse in water.^41^ Moreover, a microbubble has to shrink dramatically in order to achieve stable disproportionation; the Laplace pressure causes the shrinking and disappearance of the microbubble’s gas core. This implies that during this time period some rearrangement of the particles adsorbed on the microbubble surface and/or further adsorption of particles (SiQD) to the microbubbles can occur, forming a more closely packed particle layer necessary for long-term stability.^41^ Furthermore, apart from being a very good stabilization agent, the photoluminescent functionalized SiQD are well known for their biomedical applications due to their outstanding photoluminescence quantum yield, photostability, biocompatibility with living cells, and good water dispersibility, making them excellent for intracellular probes and biomarker/cell-imaging contrast agents that enable microbubble imaging in both fluorescence and ultrasound. Future work developing carefully designed SiQD-loaded microbubbles with a diameter of ∼5 μm has the potential of providing the contrast for photoacoustic imaging as a complementing technique to ultrasound imaging, in addition to being a potential tracker for controlled drug delivery.

This work shows that the stability of the microbubbles undergoes significant improvement due to the use of the triple T-junction coating process and the SiQD nanoparticles. Enhanced stability is achieved by SiQD nanoparticles “jamming” the liquid–gas interface, which arrests the shrinkage of microbubbles, a phenomenon known as the “jamming effect”.^42^ Moreover, the stability is also enhanced by manipulating the shell composition; the addition of SiQD to the formulation is expected to increase the resistance to bubble dissolution, which works together with the jamming effect to resist bubble shrinkage and deterioration.^43^ Improving microbubble stability is of substantial interest owing to its importance in biomedical and food applications. In particular, for bio-imaging/contrast agent applications, the lifetime of the microbubbles dictates the time period over which resolution and diagnostic information can be achieved.^44^ Microbubble stability is also an important consideration in drug delivery—any shrinkage owing to gas diffusion from the bubbles is expected to damage their drug release efficacy, whereas the destruction of shell substance can cause premature discharge of the encapsulated substance.^45^

## Conclusions

Monodisperse SiQD-loaded BSA microbubbles down to 22.8 ± 1.4 μm in diameter were generated through a new microfluidic setup comprising three T-junctions in series with 100 μm capillaries. We compare and demonstrate enhanced stability of microbubbles generated by triple T-junction and SiQD loading in the microbubble shell. The microbubble diameter and stability in air were varied by changing T-junction numbers, capillary diameter, liquid flow rate, BSA concentration, and SiQD concentration. Computational modeling of microbubble diameter and stability agreed with experimental data. Fluorescence microscopy confirmed the integration of SiQD on the microbubble surface, which retained the same morphology as those without SiQD. Microbubbles prepared with SiQD showed greater stability than those without, and the lifetime of microbubbles increased with SiQD concentration. The present research, for the first time, sheds light on a potential new route employing up to three T-junction setups to form stable, monodisperse, multi-layered, and well-characterized protein and quantum dot-based protein microbubbles with enhanced stability. Extending to beyond three T-junctions is likely to be cumbersome in an engineering sense and also involves microbubble fission, which demands a different operating model.
